# A prime/boost DNA/Modified vaccinia virus Ankara vaccine expressing recombinant *Leishmania* DNA encoding TRYP is safe and immunogenic in outbred dogs, the reservoir of zoonotic visceral leishmaniasis

**DOI:** 10.1016/j.vaccine.2008.11.094

**Published:** 2009-02-11

**Authors:** Connor Carson, Maria Antoniou, Maria Begoña Ruiz-Argüello, Antonio Alcami, Vasiliki Christodoulou, Ippokratis Messaritakis, Jenefer M. Blackwell, Orin Courtenay

**Affiliations:** a Populations and Disease Research Group, Department of Biological Sciences, University of Warwick, Gibbet Hill Road, Coventry CV4 7AL, UK; b Laboratory of Clinical Bacteriology, Parasitology, Zoonoses and Geographical Medicine, Faculty of Medicine, University of Crete, Crete 71003, Greece; c Centro de Biología Molecular Severo Ochoa (CSIC-UAM), Campus de Cantoblanco, Nicolás Cabrera 1, 28049 Madrid, Spain; d Cambridge Institute for Medical Research, Wellcome Trust/Medical Research Council Building, Addenbrooke's Hospital, Hills Road, Cambridge CB2 2XY, UK

**Keywords:** *Leishmania infantum*, Tryparedoxin peroxidase, Prime/boost DNA/MVA vaccination

## Abstract

Previous studies demonstrated safety, immunogenicity and efficacy of DNA/modified vaccinia virus Ankara (MVA) prime/boost vaccines expressing tryparedoxin peroxidase (TRYP) and *Leishmania* homologue of the mammalian receptor for activated C kinase (LACK) against *Leishmania major* challenge in mice, which was consistent with results from TRYP protein/adjuvant combinations in non-human primates. This study aimed to conduct safety and immunogenicity trials of these DNA/MVA vaccines in dogs, the natural reservoir host of *Leishmania infantum*, followed-up for 4 months post-vaccination.

In a cohort of 22 uninfected outbred dogs, blinded randomised administration of 1000 μg (high dose) or 100 μg (low dose) DNA prime (day 0) and 1 × 10^8^ pfu MVA boost (day 28) was shown to be safe and showed no clinical side effects. High dose DNA/MVA vaccinated TRYP dogs produced statistically higher mean levels of the type-1 pro-inflammatory cytokine IFN-γ than controls in whole blood assays (WBA) stimulated with the recombinant vaccine antigen TRYP, up to the final sampling at day 126, and in the absence of challenge with *Leishmania*. TRYP vaccinated dogs also demonstrated significantly higher TRYP-specific total IgG and IgG2 subtype titres than in controls, and positive *in vivo* intradermal reactions at day 156 in the absence of natural infection, observed in 6/8 TRYP vaccinated dogs. No significant increases in IFN-γ in LACK-stimulated WBA, or in LACK-specific IgG levels, were detected in LACK vaccinated dogs compared to controls, and only 2/9 LACK vaccinated dogs demonstrated DTH responses at day 156. In all groups, IgG1 subclass responses and antigen-specific stimulation of IL-10 were similar to controls demonstrating an absence of Th2/T_reg_ response, as expected in the absence of *in vivo* restimulation or natural/experimental challenge with *Leishmania*.

These collective results indicate significant antigen-specific type-1 responses and *in vivo* memory phase cellular immune responses, consistent with superior potential for protective vaccine immunogenicity of DNA/MVA TRYP over LACK.

## 1 Introduction

Zoonotic visceral leishmaniasis (ZVL) caused by the sandfly-borne intracellular protozoan parasite *Leishmania infantum* (=*L. chagasi*) [1] is endemic in the Mediterranean basin, South America and parts of Asia, and is recognised as a re-emerging disease by the World Health Organization. Development of a vaccine for ZVL in the reservoir host, the domestic dog, has been identified as a research priority by WHO/TDR [2], and mathematical models have highlighted canine vaccination as potentially the most practical and effective means of disease control in humans [3,4]. The only commercially available *Leishmania* vaccine (Leishmune^®^) is based on a purified parasite preparation, and is only licensed for use in dogs in Brazil [5]. Although trials in naturally exposed Brazilian dogs showed 80% vaccine efficacy [6], transient adjuvant-related side effects such as anorexia and local pain/swelling [7] may reduce uptake and compliance among vets and dog owners. Development of additional novel vaccine candidates is advisable, since the next generation of vaccines/vaccine antigens should always be waiting in the wings, and we should continue to improve on methods of delivery that will safely elicit lasting immunological memory. Experimental DNA vaccines are the subject of increasing numbers of human and veterinary clinical trials, since they elicit the T-cell memory required for long term protection [8], are extremely safe, easy to standardize, and are highly stable for storage and distribution purposes in tropical environments where cold chain may be unavailable [9].

Analysis of expressed sequence tags from cDNA libraries of *Leishmania major* [10] led to the discovery and functional characterisation [11] of tryparedoxin peroxidase (TRYP, also known as thiol specific antioxidant or TSA [12]), which plays a role in protection of the parasite from oxidative damage. TRYP is tandemly repeated and highly conserved across *Leishmania* spp. (91% amino acid identity with *L. infantum*), highly represented in cDNAs libraries from promastigotes [10], and highly expressed at mRNA level in promastigotes and amastigotes [13]. DNA alone or DNA/modified Vaccinia virus Ankara (MVA) prime/boost vaccine delivery highlighted TRYP as a highly effective inducer of protective immunity against virulent challenge with *Leishmania major* in susceptible BALB/c mice as shown by reduction in footpad lesion size following injection of promastigotes at 16 weeks post-vaccination [14]. These findings are consistent with studies using TRYP protein/adjuvant combinations in mice and non-human primates [15]. DNA/recombinant Vaccinia virus heterologous prime/boost vaccine protocols are now known to be superior to homologous challenge with DNA, since they stimulate more robust and longer lived synergistic cellular immune responses [16]. In mice it has been demonstrated that although both DNA/DNA and prime/boost DNA/MVA vaccines expressing TRYP protected against *L. major* challenge in the effector phase (2 weeks post-boost), the protection induced by prime/boost TRYP delivery was superior in the memory phase (16 weeks post-boost) [17], possibly due to stimulation of CD8+ T cells which are now recognised as an important element in maintenance of vaccine induced memory [18]. Importantly, TRYP was shown to be far superior as a protective vaccine to the previously described *Leishmania* homologue of the receptor for activated C kinase (LACK) [19], the functional correlate for this being higher IL-10 from regulatory T cells elicited by LACK and a higher IFN-γ:IL-10 ratio associated with TRYP (indicative of a type-1 pro-inflammatory response driven by IFN-γ secreting Th1-type CD4+ cells) compared to LACK vaccination [14]. To date, no research has been published describing the immunological responses of dogs to DNA/MVA TRYP as a potential vaccine against ZVL.

In dogs, previous research has shown that a prime/boost vaccine employing the replication competent Western Reserve strain vaccinia virus expressing LACK was safe and immunogenic, and induced 60% protective immunity against experimental i/v challenge infection with *L. infantum* at 2 weeks post-boost [20]. However, superior protection against infection, and higher T-cell proliferative responses were induced by a prime/boost vaccine which expressed LACK using the MVA strain [21], in line with previous murine research which showed that highly attenuated vaccinia virus strains such as MVA are associated with superior vaccine immunogenicity [22]. Research into prime/boost MVA canine vaccines is of particular importance due to safety concerns regarding unattenuated vaccinia strains such as Western reserve. MVA is also the current vaccinia virus strain of choice for human clinical investigations, having been used in over 120,000 human patients without documented adverse side effects, even in immunocompromised humans [23,24]. The DNA/MVA approach is currently being applied to development of prime/boost vaccines for humans, against HIV [25], malaria [26], tuberculosis [27] and tumours [28].

Following the previous successful safety, immunogenicity and efficacy studies of the prime/boost DNA/MVA TRYP vaccine against *L. major* in mice [14,17], this study aimed to demonstrate safety and immunogenicity of DNA/MVA TRYP and LACK in a cohort of 22 uninfected, unexposed outbred dogs followed-up for 4 months.

## 2 Materials and methods

### 2.1 Study population and experimental set-up

A cohort of 22 young (median age 18 months, range 4–24 months) uninfected outbred dogs from a ZVL endemic area (Crete, Greece) were enrolled for vaccination with DNA/MVA TRYP, LACK or control, and followed-up for 4 months post-prime/boost vaccination between June and November 2007. Dogs were recruited with informed consent from owners in villages of the Heraklion prefecture within 15 km radius of the city of Heraklion, on the criteria of being negative to all diagnostic tests: (1) Indirect immunofluorescent antibody test (IFAT) [29], (2) Crude *Leishmania* parasite antigen (CLA) ELISA [30], and (3) PCR of buffy coat to detect DNA expressing the internal transcribed spacer 1 region of the ribosomal RNA gene (ITS-1 rRNA) of *Leishmania* spp. [31]. The sample comprised 59% mixed breeds, the remainder including local breeds (Cretan/Hellenic hounds) (*n* = 4), Belgian Shepherd (*n* = 2) and pit bull terrier (*n* = 1), at a male: female ratio of 1.2:1.

Dogs were housed in pairs, or individually (adjacent and within sight of each other), in kennels located at the University Hospital of Crete, Heraklion, which were modified for the purpose to conform with EC regulatory standards and UK Home Office Code of Practice for housing of laboratory dogs [32]. Prior to commencement of trials, all dogs received routine vaccination for distemper, canine parvovirus, canine adenovirus and leptospirosis (Hexadog, Merial), in addition to oral antihelminthic treatment with praziquantel/fenbendazole (Caniquantel Plus, New Vet AE). To rule out exposure to *Leishmania* wild type during the transmission season (May–October), dogs were fitted with deltamethrin-impregnated collars (Scalibor, Intervet) and checked daily for collar loss, or treated instead with fortnightly doses of topical 10% imidacloprid/50% permethrin solution (Advantix, Bayer AG). Kennels were monitored continuously for sandfly activity by routine light trapping and sticky traps [33]. No sandflies were detected at the kennels during the trial. After completion of trials, all dogs were returned to their owners.

### 2.2 Vaccine administration

Dogs were randomized to receive intramuscular injections, from blinded operators, in the craniolateral aspect of the right quadriceps femoris, with DNA TRYP or LACK (100 μg; *n* = 4, or 1000 μg; *n* = 5), or control plasmid DNA (1000 μg; *n* = 4) on day 0, followed 28 days later by 10^8^ pfu MVA TRYP or LACK vaccine (or empty MVA vehicle as control). This prime/boost regime is similar to that employed in previous canine studies [20,21], in which administration of plasmid DNA (100 μg) and recombinant Vaccinia virus (10^7^ to 10^8^ pfu) were carried out 14 days apart. Safety and immunogenicity were measured as described below.

### 2.3 Safety

Dogs were kept under veterinary surveillance post-“prime” and “boost” to detect the occurrence of potential adverse reactions. Safety was assessed by daily clinical examinations for 4 days post-vaccination (as detailed in European Medicines Agency (EMeA) requirements [34]), with defined clinical end-points (local pain on palpation; inflammation; ulceration; alopecia; apathy; fever; diarrhoea; anorexia). Body weight was recorded weekly. Pre- and post-vaccine haematological and biochemical parameters were measured by collection of blood samples at 2 days before and 2 days after each vaccination. Blood was collected by jugular or cephalic venepuncture in 2 ml EDTA anticoagulated and plain serum gel tubes. Samples were sent by same day courier at +4 °C to a commercial laboratory (Microanalysi, Athens), and processed for routine biochemical tests (urea, creatinine, aspartate aminotransferase (AST), alanine aminotransferase (ALT), creatine phosphokinase (CPK) and total bilirubin) using a standard Aeroset dry chemistry analyzer (Abbott-Toshiba, USA) and red/white blood cell counts using a PCE-210 automatic blood cell counter (Erma Inc., Japan).

### 2.4 Immunogenicity

#### 2.4.1 Cytokine assays

Immunogenicity was assessed by measurement of cytokine levels (IFN-γ, TNF-α, and IL-10) expressed by antigen stimulated lymphocytes in whole blood assays (WBA) [35], measured pre-vaccination (day 0) and on days 26, 42, 70, 98 and 126 following first vaccination. Blood collected at time points detailed above by jugular or cephalic venepuncture in heparin anticoagulant was diluted 1:10 in RPMI supplemented with 100 IU/ml penicillin, 100 μg/ml streptomycin and 2 mM l-glutamine, and incubated in 96-well flat bottom plastic culture plates. Triplicate wells (200 μl per well) were incubated for each antigen or mitogen (TRYP, LACK, CLA and Concanavalin A: 10 μg/ml), including negative control (unstimulated) wells, for a period of 5 days at 37 °C in 5% CO_2_ in air. Supernatants from each of the three replicate wells were pooled and stored at −80 °C until required. Measurement of cytokines expressed in culture supernatants was carried out by quantitative ELISA using commercially available reagents. Duoset kits (R&D systems, UK) were used to detect IFN-γ and TNF-α, while matched pair monoclonal capture/polyclonal detection antibodies were employed for IL-10 measurement, using the supplied recombinant protein standards, according to the manufacturer's recommendations. Background levels in unstimulated control wells were deducted from antigen-stimulated values to quantify antigen-specific cytokine production (with negative values recorded as zero). The mean values for background levels of IFN-γ, TNF-α and IL-10 were 65 pg/ml (range 0–313), 47 pg/ml (range 0–527), and 162 pg/ml (range 0–982). TRYP and LACK antigens were not available to measure pre-vaccination (day 0) cytokine levels, therefore cytokine measurements for these antigens commenced from day 26 onwards.

#### 2.4.2 ELISA

Serological responses to vaccination (total specific IgG, IgG1 and IgG2 subtypes) were measured by anti-TRYP and anti-LACK ELISA in all dogs at all 6 follow-up time points (day 0–126). 96-well polystyrene microtitre plates (Maxisorp, Nunc A/S, Roskilde) were coated overnight at 4 °C with 50 μl 0.05 M carbonate/bicarbonate coating buffer, pH 9.6 (Sigma–Aldrich, UK) containing 0.5 μg TRYP or 0.25 μg LACK (prepared as described below) per well. Wells were washed three times with PBS/0.05% Tween 20 (repeated between each step detailed below). Blocking was performed with 2% dried milk powder in carbonate/bicarbonate buffer for 2 h at 37 °C, and 50 μl of the appropriate dilution of dog serum in PBS/0.05% Tween20/2% dried milk powder was added to each well. All samples were run in duplicate. For detection of total IgG, 50ul of anti-dog IgG conjugated to horseradish peroxidase (HRP) (Sigma–Aldrich) was used at 1:1000 dilution for 1 h incubation at 37 °C, while for antibody subtyping, goat anti-IgG1-HRP conjugate at 1:500 dilution, or sheep anti-IgG2-HRP conjugate at 1:10,000 dilution (Bethyl Laboratories, Montgomery, TX, USA) were added. 100 μl substrate solution (Tetramethylbenzidine (TMB); Sigma–Aldrich, UK) was then added, the reaction was stopped after 20 min incubation at room temperature using 50 μl 0.5 M H_2_SO_4_, and the optical density of reaction product was read using an automated ELISA plate reader (Multiskan EX, Thermo Fisher, UK) set at 450 nm. Positive and negative controls were included on each plate. The sample-to-positive ratio (s/p) [36] for each sample was calculated as the mean raw absorbance at 450 nm of duplicate test samples relative to a highly positive reference positive sample (from a parasitologically confirmed polysymptomatic Brazilian dog [30]) which was included on every ELISA plate. For subtyping experiments, to measure antigen-specific antibody titre in arbitrary units, titration curves were plotted for each serum sample using doubling dilutions from 1:100 to 1:3200 (IgG1) or alternate doubling dilutions from 1:200 to 1:204,800 (IgG2). The cut-off point was calculated as the mean s/p ratio of all dogs at time 0 (pre-vaccination). Using maximum likelihood, a straight line was fitted to the linear portion of the s/p ratio titration curve, and the reciprocal of the dilution rate at the point of intersection with the cut-off value was calculated as an estimate of antibody titre.

#### 2.4.3 Intradermal tests

Cellular immune responses *in vivo* were measured at day 156 by intradermal skin testing [37] using 0.1 μg TRYP and LACK recombinant antigen (prepared as described below) in 0.1 ml sterile pyrogen-free PBS (or 0.1 ml PBS alone, as a control) injected intradermally at the right inner thigh, a distance of 5 cm apart. The size of the indurated area was measured at 72 h after injection. Two measurements were taken at 90° to each other using vernier calipers, and the mean of the two numbers was recorded. A positive reaction was considered as >5 mm.

### 2.5 DNA and MVA vaccine preparation

Production of the DNA and MVA vaccines were carried out following GLP guidelines at the Cambridge Institute for Medical Research (DNA) and the Centro de Biología Molecular Severo Ochoa (MVA), respectively, as described in previous research [14]. Briefly, plasmid DNA was purified under sterile conditions using EndoFree Plasmid Giga kits (Qiagen) with pyrogen-free materials, and the final product resuspended in pyrogen-free PBS. Recombinant MVA expressing TRYP and LACK were originally prepared as described [14]. Purified stocks of recombinant MVA grown in RK13 cells under sterile conditions were prepared as described [38] by ultracentrifugation through a sucrose cushion, resuspended in 10 mM Tris–HCl (pH 9), stored at −80 °C until required, and diluted in pyrogen-free PBS for final inoculation. Expression of protein from recombinant MVA-infected culture lysate was checked by Western blotting using sera from DNA-vaccinated mice, demonstrating the expected protein bands at 22 kDa for TRYP and 18 kDa for LACK.

### 2.6 TRYP, LACK and CLA antigen preparation

Recombinant proteins used for *in vitro* immunology assays, and to test intradermal reactivity *in vivo*, were prepared by Novexin Ltd. (Babraham, UK) under GLP using constructs originally prepared by the Cambridge lab [14] by cloning TRYP or LACK into the expression vector pET-15b (Novagen) and transformation into *Escherichia coli* BL21 (DE3) host cells. Recombinant protein was purified by affinity column chromatography using 1 ml HisTrap FF columns (GE Healthcare). Immobilised target proteins were washed with buffer containing NV polymer to dissociate and remove endotoxin contamination before being eluted with 10 mM Tris–HCl (pH 8.5), 0.5 M NaCl and 250 mM imidazole, and desalted into low-LPS PBS using PD10 desalting columns (GE Healthcare). Proteins were diluted in pyrogen-free PBS for intradermal inoculation into dogs. Crude freeze-thawed *Leishmania infantum* CLA was prepared from stationary phase promastigotes as described previously [14].

### 2.7 Statistical analysis

Comparison of mean cytokine levels in quantitative ELISAs, and antibody titres in IgG subtyping experiments, was performed using non-parametric Wilcoxon rank sum tests. Differences between vaccine group biochemical and haematological parameters were tested for using one-way ANOVA, with Scheffe multiple comparison tests where appropriate. Statistical significance was set at *P* < 0.05. All analyses were carried out in STATA v9.

### 2.8 Ethics

Trials were undertaken to confirm safety in the target population of genetically diverse outbred dogs following EMeA scientific guidelines for veterinary medicinal products [34,39], EEC directive 86/609/EEC [40] and with approval from local government. Dogs were cared for by fully trained animal house staff under veterinary supervision. Kennels were approved by Hellenic Government Veterinary Officers (Document ref: 4381) and compliance with relevant legal requirements under Greek laws (160/1991) relating to animal welfare certified by the Hellenic Republic Ministry of Rural Development & Food: General Veterinary Authority K.A.F.E. Department ‘A’ (Document ref: 319083). Written informed consent was gained from dog owners prior to commencement of all trials. Animals remained the legal property of owners, and were returned after completion of the study. In the absence of a Cretan ethical committee for animal procedures, protocols conformed to the spirit of UK Home Office requirements for United Kingdom research establishments, and with ethical approval from the University of Warwick Biological Ethics Committee. Institutional approval for the use and modification of kennels for the vaccine trials was granted by the University of Crete Scientific Board (Document ref: 4/31-1-2007).

## 3 Results

### 3.1 Safety

#### 3.1.1 Clinical examination

**Fig. 1:**
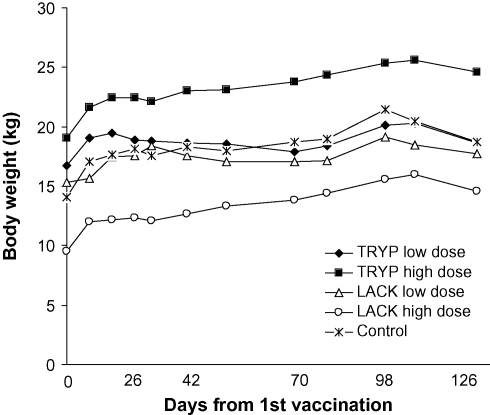
Mean body weight (kg) of vaccine and control dog groups from time of 1st vaccination.

Examination post-vaccination detected no adverse clinical side effects except transient pain on palpation of the injection site in one low dose LACK dog on the morning following second vaccination. No swelling, alopecia or systemic signs were recorded in any animal. Mean body weights of all vaccine groups increased slowly throughout the trial (Fig. 1), partly due to growth of young dogs in each group. One female animal in the TRYP low dose group was vaccinated in the early stages of gestation, before the pregnancy was apparent on clinical examination. Subsequently to discovery of the pregnancy, this bitch was monitored closely throughout an uneventful gestation, and delivered normal puppies. Data from this animal were excluded from all subsequent analyses.

#### 3.1.2 Clinical biochemistry and haematology

Between group comparison of blood biochemical (AST, ALT, creatinine, urea, total bilirubin and CPK) and haematological parameters (total red blood cell count) pre- and post-prime and boost vaccinations showed no statistically significant differences between TRYP, LACK and control groups (ANOVA; *P* ≥ 0.11). Comparison between group mean white blood cell counts at time 0 (before 1st vaccine) approached significant difference (ANOVA; *P* = 0.053), however no statistically significant differences between individual vaccine groups were identified using the Scheffe multiple comparison test, and no subsequent post-vaccine between-group differences were found (*P* ≥ 0.20).

### 3.2 Immunogenicity

#### 3.2.1 IFN-γ cytokine response

**Fig. 2:**
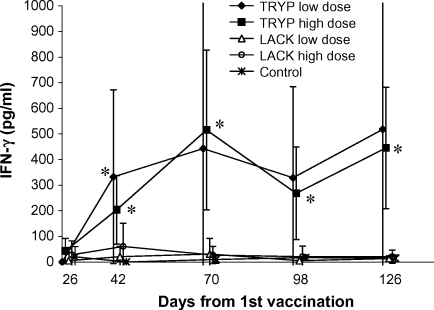
Mean IFN-γ (95% C.I.) in individual vaccine groups in TRYP WBA. IFN-γ levels were measured in whole blood cytokine stimulation assays using TRYP antigen, at the indicated time points after 1st vaccination on Day 0 with TRYP or LACK (low or high dose) DNA vaccine, or control placebo DNA. 2nd vaccination with MVA TRYP, MVA LACK or placebo (as appropriate) was carried out on Day 28. For each time point, the *x*-axis has been stretched to allow clear visualization of error bars. *Denotes a significant difference between vaccine group and control (Wilcoxon rank sum test; *P* < 0.05). One outlier point in TRYP low dose vaccine group at day 126 removed (IFN-γ = 3576 pg/ml); upper confidence limits are truncated at 1000 pg/ml on the vertical scale, for clarity.

**Fig. 3:**
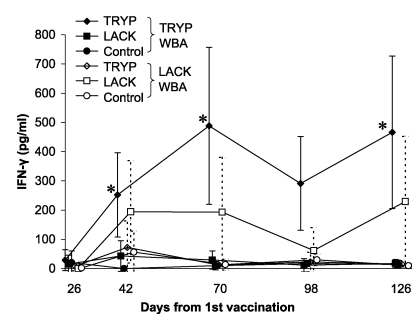
Mean IFN-γ (95% C.I.) in combined high and low dose vaccine groups in TRYP and LACK WBA. IFN-γ levels were measured in whole blood cytokine stimulation assays using TRYP and LACK antigen, at the indicated time points after 1st vaccination. Results from TRYP and LACK low and high dose vaccine groups are amalgamated. Filled points on the graph with solid error bars represent IFN-γ response to TRYP antigen stimulation in WBA, open points with dotted error bars show IFN-γ response to LACK stimulation. *Denotes a significant difference between vaccine group and control (Wilcoxon rank sum test; *P* < 0.05).

Mean IFN-γ levels in response to WBA stimulation with TRYP antigen in TRYP high dose vaccinated dogs (1000 μg DNA) were significantly higher than controls at all time points from day 42 onwards. In the TRYP low dose (100 μg DNA) group, after removal of an outlier IFN-γ value of 3576 pg/ml at day 126, vaccinated dogs showed higher mean IFN-γ levels than controls at day 42 only (Fig. 2). LACK-specific IFN-γ responses in both high and low dose LACK vaccine groups were not significantly different from controls at any time point. We did not detect any significant difference in TRYP-specific IFN-γ levels between high and low dose TRYP groups at any time point (*P* ≥ 0.27; Wilcoxon rank sum test). Similarly, no significant difference was detected between high and low dose LACK dogs’ mean IFN-γ levels, in LACK-stimulated WBA (*P* ≥ 0.45; Wilcoxon rank sum test). The combined results of high and low dose groups (Fig. 3) showed that, overall, mean IFN-γ levels in response to TRYP WBA were significantly higher in TRYP vaccinated dogs than in controls at 3/4 time points post-vaccination (*P* < 0.05: Wilcoxon rank sum test), whereas no significant difference was seen between LACK vaccinated dogs and controls in LACK WBA.

Mean IFN-γ responses to CLA antigen in all vaccine groups were consistently low (≤120 pg/ml) or below background (data not shown), showing no significant association with vaccine group.

#### 3.2.2 IL-10 cytokine response

**Fig. 4:**
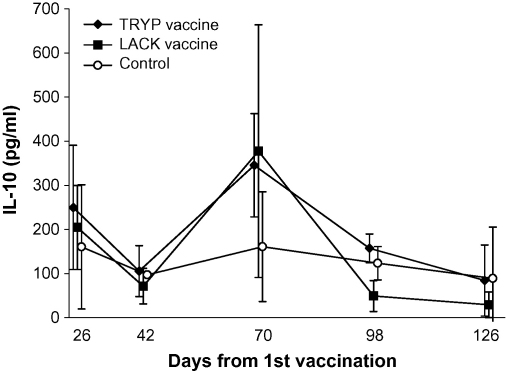
Mean IL-10 (95% C.I.) in TRYP WBA. IL-10 levels were measured in whole blood cytokine stimulation assays with TRYP antigen, at the indicated time points after 1st vaccination. Results from TRYP and LACK low and high dose vaccine groups are amalgamated.

No significant differences were observed between high and low dose TRYP (*P* ≥ 0.10) or LACK (*P* ≥ 0.09) vaccine group IL-10 responses, therefore results from the two dose rates were combined for further analysis. Mean IL-10 levels in vaccinated dogs were not significantly different from controls, showing no obvious change over time apart from a transient increase in mean IL-10 levels at Day 70 in both TRYP and LACK vaccinated dogs (not significantly different from controls: *P* ≥ 0.12; Wilcoxon rank sum test), in response to both TRYP (Fig. 4) and LACK antigens (similar results, data not shown). Mean IL-10 responses to CLA antigen were consistently low (≤62 pg/ml) or below background (data not shown), showing no significant association with vaccine group.

#### 3.2.3 TNF-α cytokine response

None of the vaccinated groups showed significant differences in mean TNF-α level compared with controls at any time point (data not shown).

#### 3.2.4 Intradermal tests

A positive skin test response to TRYP antigen (>5 mm) was observed in 4/5 TRYP high dose dogs and 2/3 TRYP low dose dogs at day 156. The TRYP low dose dog with a negative skin test result corresponded to an animal which had consistently low IFN-γ cytokine assay responses to TRYP, whereas the skin test negative animal in the high dose TRYP group paradoxically showed high IFN-γ responses to TRYP throughout the trial. In LACK vaccinated dogs, there was a positive skin test response to LACK antigen in 2/5 high dose dogs and 0/4 LACK low dose dogs.

#### 3.2.5 ELISA IgG1/IgG2 subtyping

**Fig. 5:**
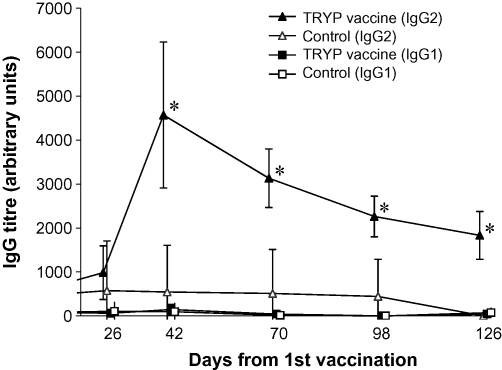
Mean TRYP-specific IgG1 and IgG2 antibody subtype titres (95% C.I.) by vaccine group. TRYP-specific IgG responses were measured at the indicated time points by ELISA using HRP conjugated antisera to detect IgG1 and IgG2 subtypes. Dogs were vaccinated with DNA TRYP or control placebo at Day 0. MVA TRYP or placebo was administered at Day 28. N.B. * denotes a significant difference between mean IgG levels in vaccinated and control dogs (Wilcoxon rank sum test: *P* < 0.05).

High TRYP-specific total IgG s/p ratios were seen in dogs post-TRYP vaccination, however LACK-specific total IgG in LACK vaccinated dogs remained at baseline levels. Measurement of TRYP-specific IgG1 and IgG2 subtypes demonstrated significantly higher levels of IgG2 in both high and low dose TRYP dogs compared to controls at all time points post-vaccination (*P* < 0.05; Wilcoxon rank sum test). No difference in IgG2 levels was detected between high and low dose TRYP dogs, therefore data were combined (Fig. 5). Data from LACK vaccinated dogs are not shown due to absence of specific antibody response in these dogs. IgG1 levels in TRYP vaccinated dogs were uniformly low, and not significantly different from controls at any time point (Fig. 5).

## 4 Discussion

This study shows that uninfected, unexposed outbred endemic dogs vaccinated with DNA/MVA TRYP prime/boost vaccine produced higher antigen-specific levels of the signature type-1 cytokine IFN-γ in whole blood cytokine stimulation assays than placebo vaccinated dogs. LACK vaccinated dogs showed a similar trend that was not statistically significant. A majority of TRYP and a minority of LACK vaccinated dogs exhibited *in vivo* delayed-type hypersensitivity responses to intradermal inoculation with the appropriate recombinant vaccine antigen at day 156, indicative of antigen-specific cellular memory recall responses. The elevated antigen-specific IFN-γ level in TRYP vaccinated dogs compares with the reported high levels of IFN-γ associated with protection in murine models against *L. major* [14,17] and *L. donovani* infection [41–43], and in dogs against *L. infantum* infection and disease [44–46], and is thus indicative of vaccine-induced protective type-1 immunity and memory phase response.

TRYP vaccinated dogs were also characterized by an IgG2 subclass dominated response, whereas IgG1 subclass levels remained low and were not significantly different to control dogs at any time point. In our hands, despite previous evidence of seroconversion to LACK antigen after DNA/MVA LACK prime/boost vaccination in murine trials [14], LACK-specific IgG did not increase measurably from baseline levels. Taking IgG2/IgG1 ratio as a proxy measure of Th1/Th2 polarization of the immune response following previous research [44,46–48], these results are further evidence of a type-1 dominated response in the TRYP vaccine group, despite some controversy over the association between canine IgG subclass ratio and protective cellular immune response [49], different to the clear patterns observed in mice [14,17]. The absence of significant IgG1 (Th2) responses in the currently described vaccinated dogs was as expected due to the absence of challenge infection or restimulation with *Leishmania* antigens, in contrast with previous canine trials of DNA/rVV prime/boost vaccines in which humoral responses were measured post-experimental challenge [20,21]. For the same reason, we detected no antigen-specific increases in IL-10 levels in either vaccine or control groups, making analysis of IFN-γ:IL-10 ratios uninformative until natural challenge experiments are conducted. In murine models vaccinated with the same TRYP vaccine, a high ratio of pre-challenge IFN-γ:IL-10 in draining lymph node cells after *in vivo* crude parasite antigen restimulation was a clear indicator of vaccine success, whereas a low ratio (due to elevated IL-10 levels) predicted failure [14]. In dogs, the existence of the Th1/Th2/T_reg_ paradigm in relation to *L. infantum* infection as observed for *L. major* in mice [50–52] is not completely resolved (as reviewed [53]), and there are conflicting results regarding the role of IL-10, with some studies demonstrating IL-10 elevation in symptomatic naturally or experimentally infected dogs [46,54], whereas other work failed to show any association between IL-10 and clinical disease [55–57].

We did not detect specific cytokine responses to CLA in the present study, however this does not preclude vaccine efficacy, as indicated elsewhere, for example in mice, where protection afforded by sterol 24-c-methyltransferase vaccine against *L. infantum* correlated with high levels of antigen-specific IFN-γ, but by comparison only very low levels IFN-γ were induced by CLA [58]. Moreover, a canine trial of HASPB1/H1 vaccine, in which lymphoproliferative responses to CLA were absent post-vaccination, subsequently demonstrated partial protection against high dose experimental challenge with *L. infantum* [59].

In conclusion we have shown that vaccination of the important reservoir host of ZVL, the domestic dog, with prime/boost DNA/MVA TRYP vaccine is free from adverse side effects and shows appropriate immunogenicity consistent with protective efficacy. The combination of *in vitro* and *in vivo* test results clearly demonstrates that DNA/MVA TRYP vaccine induces a type-1 dominated pro-inflammatory cellular immune response which is necessary for protection against *Leishmania* challenge, and that immune memory persists for at least 4 months post-vaccination in the absence of restimulation or infection. Field trials are now required to test DNA/MVA TRYP vaccine efficacy for prevention of ZVL infection and disease in naturally exposed dogs in *Leishmania* endemic areas.
